# Early eGFR fluctuation and 180-day outcome after thrombectomy for large-artery atherosclerotic stroke

**DOI:** 10.3389/fneur.2026.1872558

**Published:** 2026-09-21

**Authors:** Yao Xu, Boyan Pan, Guanao Wu, Shanshan Fu, Yefeng Cai, Yuanqi Zhao, Min Zhao

**Affiliations:** 1 The Second Clinical College of Guangzhou University of Chinese Medicine, Guangzhou, Guangdong, China; 2 Department of Neurology, The Second Affiliated Hospital of Guangzhou University of Chinese Medicine, Guangdong Provincial Hospital of Chinese Medicine, Guangzhou, Guangdong, China

**Keywords:** acute ischemic stroke, eGFR fluctuation, endovascular thrombectomy, estimated glomerular filtration rate, large-artery atherosclerosis

## Abstract

**Background:**

To investigate whether early estimated glomerular filtration rate (eGFR) fluctuation is associated with 180-day functional outcome in patients with large-artery atherosclerotic stroke undergoing endovascular thrombectomy.

**Methods:**

We conducted a multicenter retrospective cohort study of consecutive patients with acute ischemic stroke attributed to large-artery atherosclerosis who underwent endovascular thrombectomy between January 1, 2024 and December 31, 2024. Among 108 patients with 180-day follow-up, 93 had at least two actual eGFR measurements within the first 7 days and comprised the primary complete-case cohort; the full 108-patient cohort after multiple imputation was examined in a sensitivity analysis. Early eGFR fluctuation was defined as the absolute difference between the maximum and minimum eGFR values within 7 days after admission. Poor functional outcome was defined as a modified Rankin Scale score of 3–6 at 180 days.

**Results:**

In the primary cohort of 93 patients, 54 had poor functional outcomes. Greater early eGFR fluctuation was associated with poor 180-day functional outcome (adjusted OR per 1 mL/min/1.73 m2 increase, 1.078; 95% CI, 1.019–1.139; *p* = 0.008). The association was consistent in Firth penalized regression (OR, 1.068; 95% CI, 1.020–1.134; *p* = 0.002) and bootstrap internal validation. It remained significant after adjustment for the number of eGFR measurements (adjusted OR, 1.065; 95% CI, 1.006–1.127; *p* = 0.029) and in the 108-patient post-imputation sensitivity cohort (adjusted OR, 1.089; 95% CI, 1.030–1.151; *p* = 0.003). Restricted cubic spline analysis showed an overall association (*p* = 0.012) without evidence of nonlinearity (*p* = 0.471).

**Conclusion:**

Greater early eGFR fluctuation was associated with poor 180-day functional outcome after thrombectomy in patients with large-artery atherosclerotic stroke. Early eGFR fluctuation may represent an exploratory prognostic marker that requires prospective external validation.

## 1 Introduction

Acute ischemic stroke (AIS) remains a leading cause of mortality and long-term disability worldwide (1). Over the past decade, endovascular thrombectomy (EVT) has substantially improved functional outcomes in patients with large-vessel occlusion, as demonstrated in multiple randomized controlled trials (2–4). Nevertheless, despite high rates of successful reperfusion, considerable heterogeneity in post-EVT recovery persists, suggesting that factors beyond angiographic recanalization critically influence neurological outcome (5).

Large-artery atherosclerosis (LAA) stroke represents a clinically important etiological subtype among EVT-treated patients (6). Individuals with LAA stroke frequently exhibit a high burden of vascular comorbidities, including hypertension, diabetes mellitus, and chronic kidney disease (7). In addition, EVT procedures in this population are often technically complex, involving prolonged procedural duration, repeated contrast exposure, and adjunctive endovascular interventions (8). Collectively, these features may impose substantial hemodynamic and inflammatory stress on vulnerable organ systems, particularly the kidney.

Renal dysfunction has consistently been associated with adverse prognosis after AIS. Most prior studies have focused on baseline renal impairment, typically assessed by estimated glomerular filtration rate (eGFR), or on dichotomous definitions of contrast-associated acute kidney injury (AKI). However, renal function during the acute phase of stroke is inherently dynamic. EVT-treated patients are exposed to peri-procedural stressors such as contrast administration (9), blood pressure fluctuations (10), systemic inflammatory activation (11), and ischemia–reperfusion injury, all of which may induce short-term instability in renal function. Dynamic renal fluctuation may therefore capture systemic physiological vulnerability beyond static baseline eGFR or threshold-based AKI definitions.

In nephrology populations, variability in eGFR over time has been linked to adverse clinical outcomes (12). In contrast, the prognostic relevance of short-term renal functional fluctuation during the acute hospitalization period after EVT remains insufficiently characterized, particularly in patients with LAA-related AIS. Whether early dynamic changes in eGFR provide incremental prognostic information beyond baseline renal function and established stroke severity markers is unknown.

Accordingly, we investigated whether early eGFR fluctuation, defined as the difference between the maximum and minimum eGFR values within the first 7 days after admission, was associated with 180-day functional outcome in patients with LAA-related AIS undergoing EVT. We hypothesized that greater early eGFR fluctuation would be associated with an increased likelihood of poor long-term functional outcome.

## 2 Methods

### 2.1 Study design and participants

We conducted a retrospective cohort study of consecutive patients aged 18 years or older with AIS attributed to LAA who underwent EVT at Guangdong Provincial Hospital of Chinese Medicine and its four affiliated hospitals between January 1, 2024 and December 31, 2024. AIS was confirmed by computed tomography (CT) or magnetic resonance imaging (MRI). Stroke etiology was classified as LAA according to the Trial of Org 10,172 in Acute Stroke Treatment (TOAST) criteria (13). Calculation of early eGFR fluctuation required at least two actual eGFR measurements within the first 7 days. Therefore, patients with at least two measurements were included in the primary complete-case analysis. Patients with only one eGFR measurement were retained in the full-cohort sensitivity analysis, in which missing eGFR fluctuation values were handled using multiple imputation. The patient selection process is shown in Figure 1, and the study was reported in accordance with the Strengthening the Reporting of Observational Studies in Epidemiology (STROBE) guidelines.

**Figure 1:**
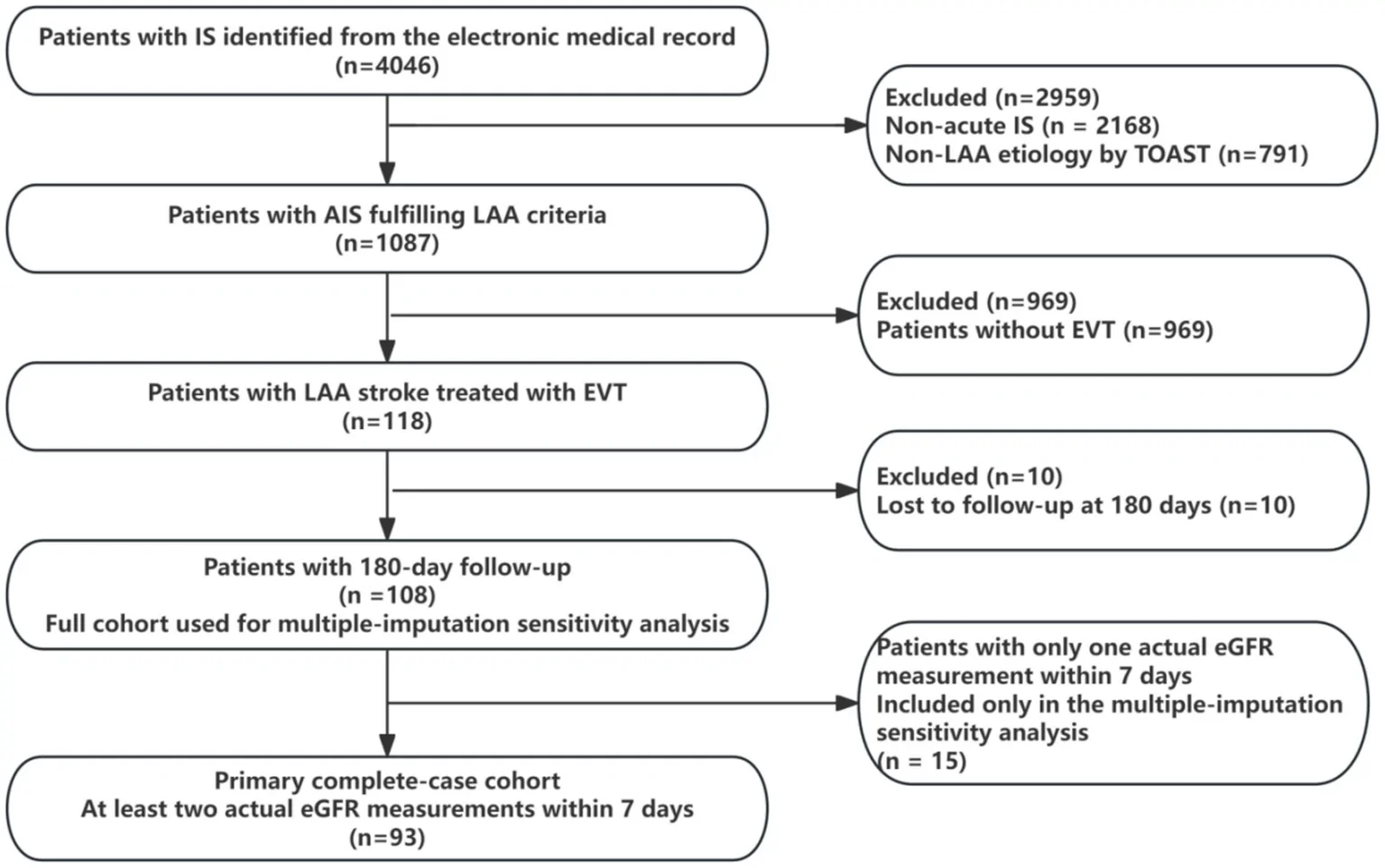
Flowchart of patient selection. Among 108 patients with 180-day follow-up, 93 with at least two actual eGFR measurements formed the primary cohort; 15 with one measurement were included only in the 108-patient post-imputation sensitivity analysis.

### 2.2 Data collection and variable definitions

Clinical data were retrospectively extracted from electronic medical records. Collected variables included demographic characteristics (age and sex), vascular risk factors and comorbidities (history of stroke or transient ischemic attack, hypertension, diabetes mellitus, hyperlipidemia, atrial fibrillation, smoking, and alcohol consumption), baseline National Institutes of Health Stroke Scale (NIHSS) score, site and territory of vessel occlusion, intravenous thrombolysis, and key EVT-related metrics (onset-to-puncture time, puncture-to-reperfusion time, procedure duration, first-pass effect, anesthesia type, and final modified Thrombolysis in Cerebral Infarction (mTICI) grade). Successful reperfusion was defined as final mTICI 2b-3.

Baseline laboratory measures obtained at admission included white blood cell count (WBC), fasting glucose, lipid profile, D-dimer, serum creatinine, and eGFR. eGFR was calculated using the Chronic Kidney Disease Epidemiology Collaboration (CKD-EPI) 2009 creatinine-based equation and expressed as mL/min/1.73 m^2^. The CKD-EPI 2009 equation was routinely used by the clinical laboratories of all five participating hospitals during the study period. Baseline eGFR was defined as the first available eGFR measurement at admission.

Early eGFR fluctuation was defined as the absolute difference between the maximum and minimum eGFR values recorded from admission through day 7, including the baseline preprocedural measurement. The primary analysis treated early eGFR fluctuation as a continuous variable, with effect estimates reported per 1 mL/min/1.73 m^2^ increase. For exploratory analyses, early eGFR fluctuation was categorized according to its distribution in the 93-patient primary cohort as T1, ≤9.8967 mL/min/1.73 m^2^; T2, >9.8967 to ≤18.8367 mL/min/1.73 m^2^; and T3, >18.8367 mL/min/1.73 m^2^. Each tertile included 31 patients. The ROC/Youden cutoff analysis was also considered exploratory and was not interpreted as a clinical decision threshold.

To explore the direction of renal function change, maximum eGFR decline from baseline was defined as baseline eGFR minus the minimum eGFR within 7 days, with negative values set to zero. Maximum eGFR increase from baseline was defined as the maximum eGFR within 7 days minus baseline eGFR, also with negative values set to zero. Percentage decline and increase were calculated as the corresponding absolute change divided by baseline eGFR and multiplied by 100. These directional analyses were exploratory.

Acute kidney injury was not formally classified because serial serum creatinine values were not obtained at prespecified, time-stamped intervals and urine-output data were unavailable. Consequently, transient changes within the KDIGO 48-h and 7-day diagnostic windows could not be assessed reliably.

### 2.3 Outcome measures

The primary outcome was poor functional outcome at 180 days, defined as a modified Rankin Scale (mRS) score of 3–6. Outcomes were assessed by trained investigators using a structured, standardized telephone-based mRS interview. The outcome assessors were unaware of the patients’ eGFR fluctuation values and were not involved in their calculation. Deaths were first identified through telephone contact with family members and were subsequently verified using relevant medical records or supporting documentation to determine the date of death. Deceased patients were assigned an mRS score of 6.

### 2.4 Statistical analyses

Continuous variables are presented as mean ± standard deviation (SD) or median with interquartile range (IQR), as appropriate, and categorical variables as counts and percentages. Baseline characteristics were compared between favorable and poor 180-day outcome groups using the Wilcoxon rank-sum test for continuous variables and the χ^2^ test or Fisher’s exact test for categorical variables, as appropriate. Exploratory comparisons across early eGFR fluctuation tertiles were performed using the Kruskal–Wallis test for continuous variables and the χ^2^ test or Fisher’s exact test for categorical variables, as appropriate. The primary association was evaluated in the 93-patient complete-case cohort using multivariable logistic regression with continuous eGFR fluctuation as the exposure. To reduce overfitting, a parsimonious model was specified on clinical grounds and included eGFR fluctuation, age, baseline NIHSS score, baseline eGFR, and successful reperfusion. The model contained five regression parameters and 54 poor functional outcomes, corresponding to 10.8 events per parameter. Firth penalized logistic regression and 1,000 bootstrap resamples were used to assess small-sample bias and internal stability.

Sensitivity analyses additionally adjusted for the actual number of eGFR measurements and separately added anesthesia type, intravenous thrombolysis, fasting glucose, and D-dimer to the parsimonious model. To further address potential measurement-frequency bias, the primary model was repeated after restricting the analysis to patients with at least three observed eGFR measurements within the first 7 days. D-dimer was entered continuously on its original scale (mg/L FEU), consistent with the final analysis files. These covariates were added in separate models to limit model complexity.

Restricted cubic spline analysis used three knots at the 10th, 50th, and 90th percentiles of early eGFR fluctuation (2.604, 13.420, and 30.184 mL/min/1.73 m^2^) to assess the functional form of the continuous association. The ROC analysis reported the AUC with 95% CI, sensitivity, and specificity; the Youden-derived cutoff and tertile analyses were designated exploratory. An adjusted proportional-odds ordinal logistic regression was performed using the complete 180-day mRS distribution from 0 to 6. Directional eGFR changes were examined as exploratory analyses, with upward and downward changes analyzed separately. Each directional measure was evaluated using the same parsimonious covariate set as the primary model, with Firth penalized logistic regression used to assess small-sample robustness.

Missingness was assessed for all collected variables. The primary analysis included the 93 patients with at least two actual eGFR measurements within 7 days, allowing early eGFR fluctuation to be directly calculated. In the original 108-patient dataset, 15 patients had missing eGFR fluctuation because only one measurement was available. Before imputation, fasting glucose was missing in 13 patients, D-dimer in 3, ALT in 1, AST in 1, and puncture-to-reperfusion time in 6. Missing covariate data were handled using multiple imputation before model fitting. Statistical analyses were performed using SPSS version 27.0 and R version 4.5.1. A two-sided *p* value <0.05 was considered statistically significant.

### 2.5 Ethics

The study was approved by the Ethics Committee of Guangdong Provincial Hospital of Traditional Chinese Medicine (Approval No. YE2025-066) and conducted in accordance with the principles of the Declaration of Helsinki. The requirement for written informed consent was waived by the Ethics Committee owing to the retrospective nature of the study.

## 3 Results

### 3.1 Baseline characteristics

A total of 118 patients met the inclusion criteria. After exclusion of 10 patients lost to 180-day follow-up, 108 patients remained. Of these, 93 had at least two actual eGFR measurements within the first 7 days and constituted the primary analysis cohort; 54 had poor functional outcomes and 39 had favorable outcomes. The remaining 15 patients had only one eGFR measurement and were included only in the full-cohort imputation sensitivity analysis. In the primary cohort, the median number of eGFR measurements within 7 days was 4 (IQR, 2–5; range, 2–7). Patients with poor outcomes underwent more measurements than those with favorable outcomes (median, 4 [IQR, 3–6] vs. 3 [IQR, 2–4]; *p* = 0.002). Measurement coverage was 100.0% at baseline, 93.5% at approximately 24 h, 52.7% at 48–72 h, and 69.9% for at least one measurement during days 4–7 (Supplementary Table S2). Baseline characteristics according to 180-day functional outcome are presented in Table 1.

**Table 1:** Baseline characteristics of the 93-patient primary cohort according to 180-day functional outcome.

Variables	Total (*n* = 93)	Favorable (*n* = 39)	Poor (*n* = 54)	*P*
Demographics				
Age, years	71.00 (61.00, 76.00)	69.00 (59.00, 73.00)	71.00 (67.00, 77.00)	0.053
Male sex	58 (62.4)	23 (59.0)	35 (64.8)	0.566
Vascular risk factors				
Prior TIA or stroke	20 (21.5)	8 (20.5)	12 (22.2)	0.843
Diabetes mellitus	29 (31.2)	11 (28.2)	18 (33.3)	0.598
Hypertension	63 (67.7)	23 (59.0)	40 (74.1)	0.124
Atrial fibrillation	25 (26.9)	10 (25.6)	15 (27.8)	0.819
Hyperlipidemia	14 (15.1)	5 (12.8)	9 (16.7)	0.609
Smoking history	30 (32.3)	12 (30.8)	18 (33.3)	0.794
Alcohol use	11 (11.8)	5 (12.8)	6 (11.1)	1.000
Site of vessel occlusion				
MCA M1 segment	30 (32.3)	18 (46.2)	12 (22.2)	0.139
MCA M2 segment	16 (17.2)	7 (17.9)	9 (16.7)	
Internal carotid artery	23 (24.7)	7 (17.9)	16 (29.6)	
Basilar or vertebral artery	20 (21.5)	6 (15.4)	14 (25.9)	
Posterior cerebral artery	4 (4.3)	1 (2.6)	3 (5.6)	
Procedural characteristics				
Baseline NIHSS score	13.00 (7.00, 18.00)	8.00 (3.00, 16.50)	15.00 (10.25, 20.00)	0.008
Onset-to-puncture time, min	254.00 (180.00, 333.00)	254.00 (234.50, 343.50)	232.50 (175.75, 293.75)	0.111
Door-to-reperfusion time, min	233.00 (196.00, 306.00)	233.00 (197.50, 323.00)	233.00 (179.75, 291.00)	0.737
Door-to-puncture time, min	147.00 (111.00, 185.00)	153.00 (141.00, 208.00)	141.00 (106.25, 181.75)	0.066
Puncture-to-reperfusion time, min	66.00 (45.00, 110.00)	60.00 (41.50, 99.00)	82.00 (51.75, 135.00)	0.065
Procedure duration, min	131.00 (90.00, 190.00)	120.00 (72.50, 182.50)	154.50 (120.00, 202.50)	0.032
Intravenous thrombolysis	23 (24.7)	12 (30.8)	11 (20.4)	0.251
First-pass effect	15 (16.1)	8 (20.5)	7 (13.0)	0.329
Successful reperfusion (final mTICI 2b-3)	88 (94.6)	38 (97.4)	50 (92.6)	0.395
General anesthesia	20 (21.5)	9 (23.1)	11 (20.4)	0.754
Laboratory findings				
WBC, ×10^9^/L	10.53 (8.20, 12.79)	9.19 (6.75, 11.18)	11.14 (8.93, 13.07)	0.007
NLR	6.04 (4.20, 9.57)	5.02 (3.30, 8.38)	6.73 (5.28, 9.85)	0.013
Platelets, ×10^9^/L	206.00 (165.00, 259.00)	222.00 (175.50, 278.50)	196.00 (160.50, 252.75)	0.224
ALT, U/L	17.00 (11.00, 26.00)	18.00 (12.50, 25.00)	17.00 (11.00, 28.50)	0.761
AST, U/L	22.00 (18.00, 32.00)	21.00 (18.50, 30.00)	24.00 (18.25, 32.00)	0.253
Creatinine, μmol/L	79.00 (65.00, 95.00)	77.00 (58.50, 91.00)	84.50 (70.25, 95.75)	0.139
Urea nitrogen, mmol/L	5.10 (3.96, 6.18)	4.67 (3.67, 5.84)	5.38 (4.29, 6.64)	0.073
Baseline eGFR, mL/min/1.73 m^2^	76.86 (63.47, 89.55)	83.61 (70.55, 95.36)	73.22 (60.82, 87.19)	0.052
LDL-C, mmol/L	2.50 (1.77, 3.07)	2.53 (1.90, 3.25)	2.48 (1.60, 2.89)	0.104
Total cholesterol, mmol/L	4.06 (3.41, 4.64)	4.35 (3.52, 4.82)	4.06 (3.36, 4.45)	0.269
Fasting glucose, mmol/L	6.90 (5.77, 9.00)	6.65 (5.83, 8.00)	7.51 (5.72, 9.95)	0.071
D-dimer, mg/L FEU	2.04 (0.70, 4.65)	1.56 (0.51, 4.65)	2.12 (0.89, 5.40)	0.139
INR	1.09 (1.02, 1.17)	1.06 (0.99, 1.14)	1.12 (1.03, 1.19)	0.072

ALT, alanine aminotransferase; AST, aspartate aminotransferase; BA, basilar artery; eGFR, estimated glomerular filtration rate; FEU, fibrinogen-equivalent units; ICA, internal carotid artery; INR, international normalized ratio; LDL-C, low-density lipoprotein cholesterol; MCA, middle cerebral artery; mTICI, modified Thrombolysis in Cerebral Infarction; NIHSS, National Institutes of Health Stroke Scale; NLR, neutrophil-to-lymphocyte ratio; PCA, posterior cerebral artery; TIA, transient ischemic attack; VA, vertebral artery; WBC, white blood cell count. Data are presented as median (interquartile range) or number (percentage), as appropriate. p values were calculated using the Wilcoxon rank-sum test for continuous variables and the χ^2^ test or Fisher exact test for categorical variables, as appropriate.

Compared with patients with favorable outcomes, those with poor outcomes had higher baseline NIHSS scores (*p* = 0.008), longer procedure duration (*p* = 0.032), higher WBC counts (*p* = 0.007), and higher neutrophil-to-lymphocyte ratios (*p* = 0.013) (Table 1).

In exploratory comparisons across early eGFR fluctuation tertiles, baseline NIHSS score (*p* = 0.002), baseline eGFR (*p* = 0.043), and smoking history (*p* = 0.045) differed significantly (Supplementary Table S1).

### 3.2 Association between early eGFR fluctuation and 180-day functional outcome

At 180 days, 54 of 93 patients (58.1%) had a poor functional outcome. The complete mRS distribution was as follows: mRS 0, 15 patients; mRS 1, 12; mRS 2, 12; mRS 3, 14; mRS 4, 5; mRS 5, 9; and mRS 6, 26.

In the parsimonious primary model, greater continuous early eGFR fluctuation was associated with higher odds of poor functional outcome at 180 days (adjusted OR per 1 mL/min/1.73 m^2^ increase, 1.078; 95% CI, 1.019–1.139; *p* = 0.008). The result was consistent in Firth penalized logistic regression (OR, 1.068; 95% CI, 1.020–1.134; *p* = 0.002). Among 996 successful bootstrap resamples, the median OR was 1.073 with a 95% percentile interval of 1.033–1.146, and the direction of the association was consistent in 100% of successful resamples. The primary continuous and exploratory tertile analyses are summarized in Table 2.

**Table 2:** Association of early eGFR fluctuation with poor functional outcome (mRS 3–6) at 180 days in the 93-patient primary cohort.

ΔeGFR exposure	*N*	Poor functional outcome (mRS 3–6), *n* (%)	Adjusted OR (95% CI)	*P*
Continuous ΔeGFR (per 1 mL/min/1.73 m^2^ increase)	93	54 (58.1)	1.078 (1.019–1.139)	0.008
ΔeGFR tertiles				
T1, ≤9.8967	31	9 (29.0)	1.00 (Reference)	—
T2, >9.8967 to ≤18.8367	31	22 (71.0)	6.77 (2.08–22.08)	0.002
T3, >18.8367	31	23 (74.2)	6.87 (1.93–24.51)	0.003
P for trend				0.002

CI, confidence interval; eGFR, estimated glomerular filtration rate; mRS, modified Rankin Scale; NIHSS, National Institutes of Health Stroke Scale; OR, odds ratio. Early eGFR fluctuation was defined as the difference between the maximum and minimum eGFR values within 7 days after admission. Adjusted models included age, baseline NIHSS score, baseline eGFR, and successful reperfusion defined as final mTICI 2b-3; early eGFR fluctuation was entered as either a continuous variable or tertile category, as indicated. The continuous OR represents the effect per 1 mL/min/1.73 m^2^ increase. Tertile analyses were exploratory. *P* for trend was calculated by modeling the tertile category as an ordinal variable.

After additional adjustment for the number of actual eGFR measurements, the association remained significant (adjusted OR, 1.065; 95% CI, 1.006–1.127; *p* = 0.029); the corresponding Firth estimate was 1.054 (95% CI, 1.010–1.122; *p* = 0.011). In a complementary sensitivity analysis restricted to 68 patients with at least three observed eGFR measurements, including 45 patients with poor functional outcomes, the association remained consistent in Firth penalized logistic regression (OR per 1 mL/min/1.73 m^2^ increase, 1.054; 95% CI, 1.009–1.127; *p* = 0.013). Firth regression was emphasized in this restricted cohort because the successful-reperfusion variable showed quasi-complete separation. Similar results were observed after separately adjusting for anesthesia type (adjusted OR, 1.077; 95% CI, 1.019–1.139; *p* = 0.009), intravenous thrombolysis (adjusted OR, 1.077; 95% CI, 1.019–1.138; *p* = 0.008), fasting glucose (adjusted OR, 1.074; 95% CI, 1.015–1.136; *p* = 0.014), and D-dimer on its original scale (adjusted OR, 1.078; 95% CI, 1.019–1.139; *p* = 0.008). In the full 108-patient post-imputation sensitivity cohort, eGFR fluctuation remained associated with poor outcome (adjusted OR, 1.089; 95% CI, 1.030–1.151; *p* = 0.003). In exploratory tertile sensitivity analyses, estimates were also materially unchanged after separate adjustment for fasting glucose (T2 vs. T1: OR, 6.69; 95% CI, 2.03–22.03; *p* = 0.002; T3 vs. T1: OR, 6.18; 95% CI, 1.72–22.22; *p* = 0.005; *P* for trend = 0.004) or D-dimer (T2 vs. T1: OR, 7.20; 95% CI, 2.16–24.02; *p* = 0.001; T3 vs. T1: OR, 7.03; 95% CI, 1.95–25.31; *p* = 0.003; *P* for trend = 0.002). Detailed continuous sensitivity analyses are reported in the Results section above, and exploratory tertile sensitivity analyses are provided in Supplementary Table S4.

Restricted cubic spline analysis showed a significant overall association between continuous eGFR fluctuation and poor outcome (*P* for overall association = 0.012), with no evidence of a nonlinear component (*P* for nonlinearity = 0.471). In the adjusted ordinal analysis, greater eGFR fluctuation was associated with a shift toward a higher, worse 180-day mRS category (common OR, 1.069; 95% CI, 1.030–1.110; *p* < 0.001). Nominal-effects likelihood-ratio tests for the proportional-odds assumption were not estimable because of sparse mRS categories; the ordinal model was therefore considered supportive sensitivity evidence.

In exploratory tertile analyses, poor functional outcome occurred in 9 of 31 patients in T1, 22 of 31 in T2, and 23 of 31 in T3. Compared with T1, the adjusted ORs were 6.77 (95% CI, 2.08–22.08; *p* = 0.002) for T2 and 6.87 (95% CI, 1.93–24.51; *p* = 0.003) for T3, with *P* for trend = 0.002. These exploratory tertile results are presented in Supplementary Table S4 and Figure 2. The ROC AUC was 0.724 (95% CI, 0.618–0.831). The exploratory Youden cutoff was 10.57 mL/min/1.73 m^2^, with 81.5% sensitivity and 64.1% specificity; this cutoff was not interpreted as a clinical decision threshold. ROC results are also summarized in Supplementary Table S4. In secondary directional analyses, patients with poor outcomes had greater maximum absolute eGFR decline (median, 9.15 vs. 0.48 mL/min/1.73 m^2^; *p* = 0.003) and greater percentage decline (12.00% vs. 0.55%; *p* = 0.001). In adjusted Firth penalized regression using the same covariate set as the primary model, maximum decline remained associated with poor outcome (OR per 5 mL/min/1.73 m^2^, 1.36; 95% CI, 1.04–1.79; *p* = 0.024), as did percentage decline (OR per 10%, 1.64; 95% CI, 1.11–2.42; *p* = 0.013). In contrast, neither maximum absolute increase (OR per 5 mL/min/1.73 m^2^, 1.08; 95% CI, 0.81–1.43; *p* = 0.600) nor percentage increase (OR per 10%, 1.00; 95% CI, 0.68–1.46; *p* = 0.987) was associated with poor outcome. These findings suggest that the observed association with overall eGFR fluctuation may be driven predominantly by downward eGFR changes. Detailed directional analyses are provided in Supplementary Table S3.

**Figure 2:**
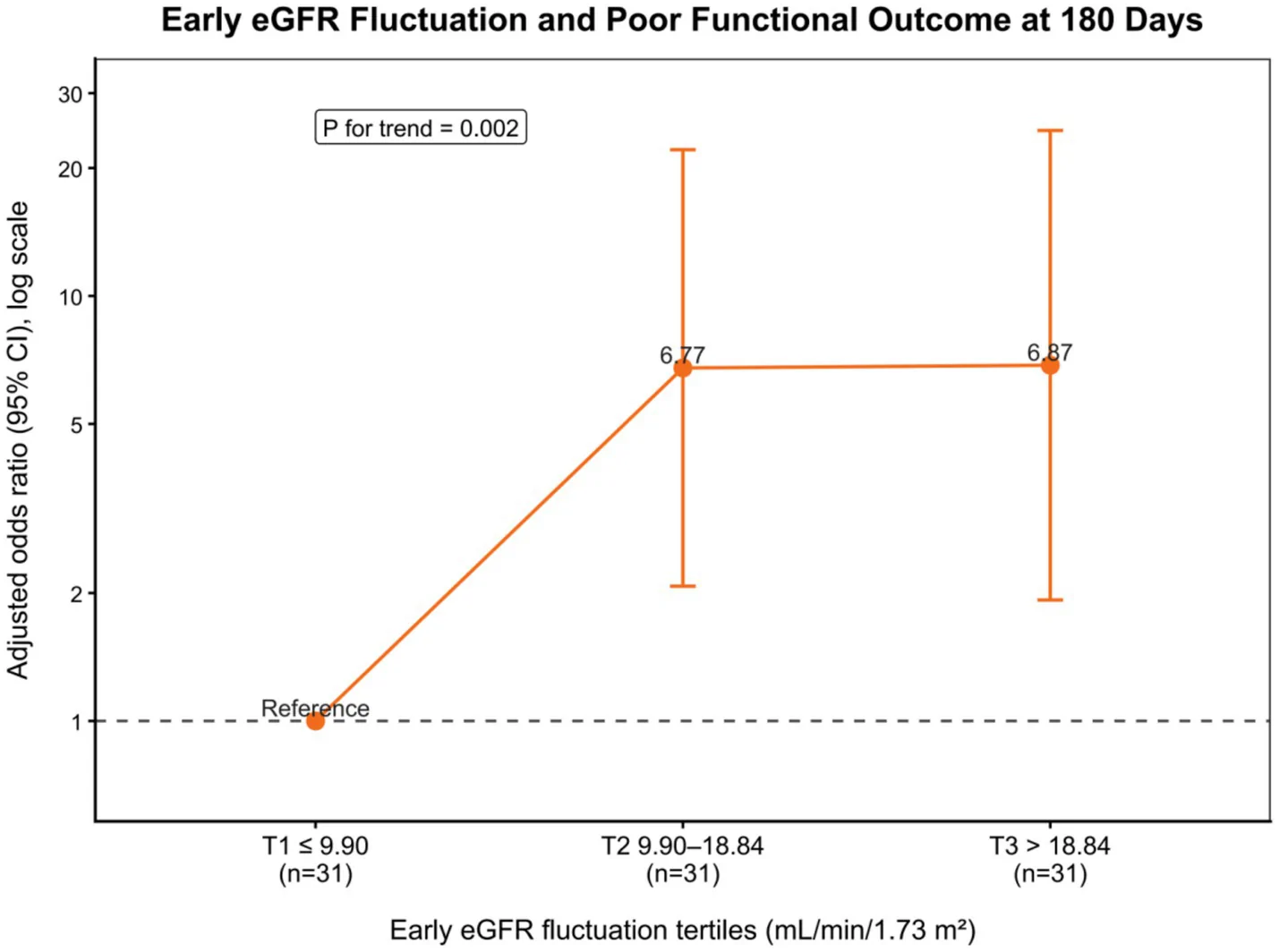
Exploratory association between tertiles of early eGFR fluctuation and poor functional outcome (mRS 3–6) at 180 days. T1 included 31 patients with 9 poor outcomes, T2 included 31 patients with 22 poor outcomes, and T3 included 31 patients with 23 poor outcomes. Odds ratios were estimated using multivariable logistic regression adjusted for age, baseline NIHSS score, baseline eGFR, and successful reperfusion defined as final mTICI 2b-3. The lowest tertile served as the reference group. Error bars represent 95% confidence intervals.

## 4 Discussion

### 4.1 Renal function and stroke prognosis

Impaired renal function has consistently been associated with adverse outcomes after AIS. Long-term data indicate that patients with eGFR <45 mL/min/1.73 m^2^ have a 1.22-fold higher risk of recurrent stroke and a 1.45-fold higher risk of death compared with those with preserved renal function (14). Similar associations have been observed in stroke-specific cohorts. In the China National Stroke Registry (CNSR) cohort published in Stroke, reduced eGFR was independently associated with increased risk of all-cause mortality and poor functional outcome, with effect modification by hypertension status (mortality OR 1.88 without hypertension; 1.80 with hypertension) (15). Similarly, in a population-based cohort stratified by diabetes status, markedly reduced eGFR conferred nearly a threefold higher risk of mortality among nondiabetic patients (OR 2.79) (16). Collectively, these findings underscore the consistent prognostic relevance of baseline renal dysfunction across diverse populations and clinical subgroups.

From a pathophysiological perspective, LAA stroke represents a manifestation of systemic atherothrombotic disease characterized by chronic vascular inflammation (17), lipid-rich plaque formation (18), and plaque instability (19), predisposing to artery-to-artery embolism or *in situ* thrombosis. Such diffuse macrovascular involvement reflects shared pathological processes affecting multiple vascular territories (20). As part of this systemic vascular burden, the renal microvasculature may likewise be exposed to similar inflammatory and atherogenic influences, as microvascular dysfunction has been described as a systemic process affecting multiple organs including the brain and kidney (21). In this context, underlying renal microvascular vulnerability may render patients particularly sensitive to physiological stress during the peri-procedural period (22). Such heightened vulnerability may manifest as short-term renal functional fluctuation. Consistent with this biological framework, early eGFR fluctuation in our LAA-related AIS cohort remained associated with 180-day functional outcome after multivariable adjustment.

### 4.2 Early eGFR fluctuation in EVT stroke

Endovascular thrombectomy has fundamentally transformed the management of AIS caused by large-vessel occlusion. Although pivotal trials such as SWIFT PRIME and REVASCAT excluded patients with severe renal impairment (23, 24), the prognostic relevance of renal function in patients undergoing EVT has subsequently been investigated in cohort studies. AKI is a clinically relevant complication after EVT and is often considered in relation to contrast exposure (25), alongside peri-procedural hemodynamic stress (26) and underlying renal vulnerability (27, 28). In a thrombectomy-treated cohort of 333 patients, severe baseline renal impairment (eGFR <30 mL/min/1.73 m^2^) strongly predicted CA-AKI (adjusted OR, 19.93; 95% CI, 2.33–170.74; *p* = 0.006), and CA-AKI was independently associated with 3-month mortality (adjusted OR, 4.68; 95% CI, 1.05–20.97; *p* = 0.04) (29). Importantly, the prognostic relevance of kidney function appears to extend beyond a binary AKI diagnosis. In a nationwide registry of 2,561 EVT-treated patients, 90-day functional independence declined as admission eGFR worsened (43.2 to 11.3%), while mortality increased (18.5–43.8%). After multivariable adjustment, end-stage renal disease (eGFR <15 mL/min/1.73 m^2^) was associated with reduced functional independence (OR, 0.35; 95% CI, 0.15–0.78), while renal impairment was associated with higher 90-day mortality, with adjusted odds ratios of 1.63 (95% CI, 1.23–2.15) for eGFR 30–59, 3.04 (95% CI, 1.70–5.43) for eGFR 15–29, and 3.74 (95% CI, 2.17–6.45) for eGFR <15 (all vs. eGFR 60–119) (30).

Most prior studies have focused on baseline renal function or overt AKI in EVT populations. In contrast, the potential clinical relevance of short-term renal fluctuation has been less clearly described. In our present study, greater early eGFR fluctuation during the first week after EVT remained associated with poor functional outcome at 180 days, even after adjustment for baseline renal function, stroke severity, and reperfusion status. Furthermore, spline analysis showed an overall association without evidence of nonlinearity, consistent with an approximately linear association rather than a distinct threshold effect.

### 4.3 Potential pathophysiological context of early eGFR fluctuation in LAA-EVT stroke

Large-artery atherosclerosis stroke reflects diffuse systemic atherosclerotic burden rather than isolated focal cerebrovascular disease (20). Systemic atherosclerotic disease is associated with increased arterial stiffness (31), while symptomatic large-vessel cerebrovascular disease may exhibit impaired cerebrovascular reactivity (32). Together, these findings are consistent with a broader atherosclerotic burden (20) and reduced cerebral autoregulatory reserve (33). The kidney occupies a unique position within this systemic vascular continuum. As a highly perfused end organ (34) supplied by both large conduit arteries and an extensive microvascular network, renal perfusion depends on intact autoregulatory mechanisms operating across multiple vascular levels (22). Disruption of renal macro and microcirculatory integrity may increase susceptibility to acute hemodynamic stress, rendering renal function particularly vulnerable to perfusion instability (35). Consequently, short-term renal functional fluctuation may be associated with systemic physiological instability, although the present study cannot establish the underlying mechanism.

In the context of EVT, this vulnerability may be further amplified. Beyond restoring focal cerebral perfusion, EVT imposes additional systemic stress through dynamic periprocedural blood pressure changes (26), inflammatory activation (11), and contrast exposure (29). In individuals with advanced atherosclerosis, the cumulative burden of ischemic and procedural stress may exceed compensatory capacity within both macrovascular and microvascular beds, predisposing to autoregulatory failure across interconnected organs.

Within this framework, early eGFR fluctuation may be associated with the combined effects of chronic atherosclerotic burden and acute physiological stress. Unlike baseline eGFR, which primarily captures static renal reserve, short-term eGFR variability may be associated with limited physiological resilience during the acute phase. These mechanistic interpretations remain hypothetical and require prospective validation.

## 5 Limitations

Several limitations should be acknowledged. First, the retrospective design and modest sample size may have introduced selection bias, residual confounding, reverse causality, and model overfitting, although a parsimonious model, Firth penalized regression, and bootstrap internal validation were used. Second, eGFR measurements were obtained according to clinical need rather than at prespecified time points. Because the maximum-minimum range is partly dependent on the number and timing of measurements, residual measurement-frequency bias cannot be completely excluded despite the consistent sensitivity analyses. Third, data on contrast volume, peri-procedural hemodynamics, dehydration, infection, nephrotoxic medication exposure, fluid therapy, and ICU care were not systematically available. AKI could not be reliably classified because serum creatinine was not measured at prespecified intervals and urine-output data were unavailable. Fourth, the ROC/Youden cutoff was derived and evaluated in the same cohort and should therefore be considered exploratory. Finally, the lack of external validation, restriction to patients with LAA stroke treated with EVT within a single regional network, and reliance on creatinine-based eGFR may limit the generalizability of our findings. Additional renal biomarkers, including cystatin C and markers of tubular or microvascular injury, were also unavailable.

## 6 Conclusion

Among patients with LAA stroke undergoing EVT, greater early eGFR fluctuation within the first week after admission was associated with poor functional outcome at 180 days. This association remained consistent across several sensitivity analyses, but the observational design does not establish causality. Early eGFR fluctuation may be a clinically accessible exploratory marker, and prospective studies with standardized renal monitoring and external validation are needed.

## Data Availability

The original contributions presented in the study are included in the article/Supplementary material, further inquiries can be directed to the corresponding author/s.
