# Supplementary material for: Early eGFR fluctuation and 180-day outcome after thrombectomy for large-artery atherosclerotic stroke
Source: Front Neurol. 2026 Sep 21;17:1872558. doi: 10.3389/fneur.2026.1872558 (PMC13634815; doi:10.3389/fneur.2026.1872558)
Supplement: Supplementary file 1 [file Table_1.DOCX]

**Supplementary Materials**

**Supplementary Table S1. Exploratory baseline characteristics according to tertiles of early eGFR fluctuation**

| **Variables** | **Total (n=93)** | **T1 (n=31)** | **T2 (n=31)** | **T3 (n=31)** | **P** |
| --- | --- | --- | --- | --- | --- |
| Demographics |  |  |  |  |  |
| Age, years | 71.00 (61.00, 76.00) | 69.00 (59.50, 73.50) | 71.00 (65.50, 76.50) | 71.00 (61.50, 78.50) | 0.535 |
| Male sex | 58 (62.4) | 16 (51.6) | 19 (61.3) | 23 (74.2) | 0.184 |
| Vascular risk factors |  |  |  |  |  |
| Prior TIA or stroke | 20 (21.5) | 7 (22.6) | 8 (25.8) | 5 (16.1) | 0.640 |
| Diabetes mellitus | 29 (31.2) | 8 (25.8) | 11 (35.5) | 10 (32.3) | 0.704 |
| Hypertension | 63 (67.7) | 19 (61.3) | 23 (74.2) | 21 (67.7) | 0.554 |
| Atrial fibrillation | 25 (26.9) | 6 (19.4) | 10 (32.3) | 9 (29.0) | 0.491 |
| Hyperlipidemia | 14 (15.1) | 5 (16.1) | 5 (16.1) | 4 (12.9) | 0.919 |
| Smoking history | 30 (32.3) | 11 (35.5) | 14 (45.2) | 5 (16.1) | 0.045 |
| Alcohol use | 11 (11.8) | 5 (16.1) | 5 (16.1) | 1 (3.2) | 0.192 |
| Site of vessel occlusion |  |  |  |  |  |
| MCA M1 segment | 30 (32.3) | 12 (38.7) | 8 (25.8) | 10 (32.3) | 0.787 |
| MCA M2 segment | 16 (17.2) | 7 (22.6) | 6 (19.4) | 3 (9.7) |  |
| Internal carotid artery | 23 (24.7) | 5 (16.1) | 9 (29.0) | 9 (29.0) |  |
| Basilar or vertebral artery | 20 (21.5) | 6 (19.4) | 6 (19.4) | 8 (25.8) |  |
| Posterior cerebral artery | 4 (4.3) | 1 (3.2) | 2 (6.5) | 1 (3.2) |  |
| Procedural characteristics |  |  |  |  |  |
| Baseline NIHSS score | 13.00 (7.00, 18.00) | 8.00 (3.00, 14.50) | 14.00 (8.00, 16.00) | 17.00 (12.00, 23.00) | 0.002 |
| Onset-to-puncture time, min | 254.00 (180.00, 333.00) | 254.00 (187.50, 351.00) | 260.00 (186.00, 347.00) | 233.00 (180.00, 287.50) | 0.509 |
| Door-to-reperfusion time, min | 233.00 (196.00, 306.00) | 233.00 (170.50, 233.00) | 240.00 (204.50, 334.50) | 233.00 (208.00, 313.50) | 0.147 |
| Door-to-puncture time, min | 147.00 (111.00, 185.00) | 147.00 (104.00, 158.00) | 147.00 (124.00, 211.00) | 153.00 (112.00, 201.50) | 0.326 |
| Puncture-to-reperfusion time, min | 66.00 (45.00, 110.00) | 72.00 (45.00, 96.00) | 90.00 (48.50, 138.00) | 60.00 (42.50, 107.50) | 0.689 |
| Procedure duration, min | 131.00 (90.00, 190.00) | 130.00 (77.50, 185.00) | 155.00 (111.00, 184.00) | 130.00 (100.00, 207.50) | 0.634 |
| Intravenous thrombolysis | 23 (24.7) | 10 (32.3) | 6 (19.4) | 7 (22.6) | 0.472 |
| First-pass effect | 15 (16.1) | 5 (16.1) | 3 (9.7) | 7 (22.6) | 0.385 |
| Successful reperfusion (final mTICI 2b-3) | 88 (94.6) | 28 (90.3) | 30 (96.8) | 30 (96.8) | 0.429 |
| General anesthesia | 20 (21.5) | 7 (22.6) | 8 (25.8) | 5 (16.1) | 0.640 |
| Laboratory findings |  |  |  |  |  |
| WBC, ×10⁹/L | 10.53 (8.20, 12.79) | 10.33 (8.79, 13.14) | 9.59 (7.37, 12.84) | 10.59 (8.66, 12.46) | 0.668 |
| NLR | 6.04 (4.20, 9.57) | 6.16 (3.58, 8.47) | 5.52 (4.47, 7.58) | 6.62 (5.21, 10.65) | 0.272 |
| Platelets, ×10⁹/L | 206.00 (165.00, 259.00) | 215.00 (171.50, 298.00) | 217.00 (162.50, 253.50) | 198.00 (161.00, 252.00) | 0.627 |
| ALT, U/L | 17.00 (11.00, 26.00) | 16.00 (11.50, 24.00) | 19.00 (11.50, 28.50) | 17.00 (10.00, 21.50) | 0.630 |
| AST, U/L | 22.00 (18.00, 32.00) | 21.00 (19.00, 28.00) | 22.00 (18.50, 32.00) | 27.00 (18.00, 38.00) | 0.417 |
| Creatinine, μmol/L | 79.00 (65.00, 95.00) | 72.00 (55.50, 96.00) | 79.00 (59.50, 90.00) | 86.00 (76.00, 100.00) | 0.060 |
| Urea nitrogen, mmol/L | 5.10 (3.96, 6.18) | 4.67 (3.79, 6.22) | 5.14 (3.58, 5.83) | 5.30 (4.29, 6.42) | 0.487 |
| Baseline eGFR, mL/min/1.73 m² | 76.86 (63.47, 89.55) | 86.77 (70.03, 96.28) | 80.79 (64.38, 90.32) | 71.23 (62.70, 80.23) | 0.043 |
| LDL-C, mmol/L | 2.50 (1.77, 3.07) | 2.50 (1.79, 3.23) | 2.40 (1.86, 2.91) | 2.50 (1.73, 3.05) | 0.786 |
| Total cholesterol, mmol/L | 4.06 (3.41, 4.64) | 3.90 (3.42, 4.75) | 4.01 (3.41, 4.36) | 4.12 (3.50, 4.82) | 0.640 |
| Fasting glucose, mmol/L | 6.90 (5.77, 9.00) | 6.90 (5.79, 7.81) | 6.90 (5.49, 9.08) | 8.26 (6.50, 10.32) | 0.058 |
| D-dimer, mg/L FEU | 2.04 (0.70, 4.65) | 1.53 (0.51, 4.65) | 1.46 (0.69, 3.09) | 2.67 (1.02, 5.95) | 0.212 |
| INR | 1.09 (1.02, 1.17) | 1.09 (0.99, 1.17) | 1.06 (1.02, 1.16) | 1.10 (1.04, 1.19) | 0.490 |

Data are median (IQR) or n (%). Early eGFR fluctuation tertiles were T1, ≤9.8967; T2, >9.8967 to ≤18.8367; and T3, >18.8367 mL/min/1.73 m². P values use the Kruskal-Wallis test for continuous variables and the χ² test or Fisher exact test for categorical variables, as appropriate. Tertile comparisons were exploratory.

**Supplementary Table S2. Number and timing of eGFR measurements in the 93-patient primary cohort**

| **Item** | **Overall** | **Favorable outcome** | **Poor outcome** | **Statistic** | **P** |
| --- | --- | --- | --- | --- | --- |
| Number of eGFR measurements, median (IQR; range) | 4 (2-5; 2-7) | 3 (2-4; 2-7) | 4 (3-6; 2-7) | Wilcoxon rank-sum | 0.002 |
| Baseline coverage | 93/93 (100.0%) |  |  |  |  |
| Approximately 24-hour coverage | 87/93 (93.5%) |  |  |  |  |
| 48-72-hour coverage | 49/93 (52.7%) |  |  |  |  |
| At least one measurement on days 4-7 | 65/93 (69.9%) |  |  |  |  |
| Testing count by tertile | T1: 2 (2-3); T2: 4 (3-5); T3: 5 (4-6) |  |  | Kruskal-Wallis | <0.001 |

**Supplementary Table S3. Exploratory directional eGFR changes according to functional outcome**

| **Metric** | **Overall, median (IQR)** | **Favorable, median (IQR)** | **Poor, median (IQR)** | **P** |
| --- | --- | --- | --- | --- |
| Maximum decline, mL/min/1.73 m² | 3.51 (0.00-13.20) | 0.48 (0.00-6.53) | 9.15 (0.00-16.47) | 0.003 |
| Relative decline, % | 5.17 (0.00-19.04) | 0.55 (0.00-8.06) | 12.00 (0.00-29.86) | 0.001 |
| Maximum increase, mL/min/1.73 m² | 5.97 (0.00-13.27) | 4.89 (0.34-10.41) | 7.38 (0.00-13.42) | 0.476 |
| Relative increase, % | 8.24 (0.00-20.49) | 5.33 (0.30-15.13) | 11.75 (0.00-21.34) | 0.526 |

Maximum decline was baseline eGFR minus the minimum eGFR within 7 days. Maximum increase was the maximum eGFR within 7 days minus baseline eGFR. Negative values were set to zero.

Adjusted Firth models: maximum decline, OR 1.36 per 5 mL/min/1.73 m² (95% CI, 1.04-1.79; P=0.024); percentage decline, OR 1.64 per 10% (95% CI, 1.11-2.42; P=0.013); maximum increase, OR 1.08 per 5 mL/min/1.73 m² (95% CI, 0.81-1.43; P=0.600); percentage increase, OR 1.00 per 10% (95% CI, 0.68-1.46; P=0.987). All models used the same parsimonious covariate set as the primary model.

**Supplementary Table S4. Exploratory tertile, ROC/Youden, and sensitivity analyses**

Panel A. Exploratory tertile analysis of early eGFR fluctuation

| **Exposure category** | **N** | **Poor outcomes, n (%)** | **Adjusted OR** | **95% CI** | **P** |
| --- | --- | --- | --- | --- | --- |
| T1, ≤9.8967 | 31 | 9 (29.0) | 1.00 | Reference |  |
| T2, >9.8967 to ≤18.8367 | 31 | 22 (71.0) | 6.77 | 2.08-22.08 | 0.002 |
| T3, >18.8367 | 31 | 23 (74.2) | 6.87 | 1.93-24.51 | 0.003 |
| Tertile trend |  |  | 2.75 per tertile | 1.44-5.23 | 0.002 |

Panel B. Exploratory ROC/Youden analysis in the 93-patient primary cohort

| **AUC** | **95% CI** | **Youden cutoff** | **Sensitivity** | **Specificity** | **Interpretation** |
| --- | --- | --- | --- | --- | --- |
| 0.724 | 0.618-0.831 | 10.57 mL/min/1.73 m² | 81.5% | 64.1% | Exploratory; not a clinical decision threshold |

Panel C. Tertile sensitivity analyses after separate adjustment for fasting glucose and D-dimer

| **Additional covariate** | **Comparison** | **Adjusted OR** | **95% CI** | **P** | **P for trend** |
| --- | --- | --- | --- | --- | --- |
| Fasting glucose | T2 vs T1 | 6.69 | 2.03-22.03 | 0.002 | 0.004 |
| Fasting glucose | T3 vs T1 | 6.18 | 1.72-22.22 | 0.005 | 0.004 |
| D-dimer | T2 vs T1 | 7.20 | 2.16-24.02 | 0.001 | 0.002 |
| D-dimer | T3 vs T1 | 7.03 | 1.95-25.31 | 0.003 | 0.002 |

T1 was the reference category. These analyses were exploratory and used the parsimonious primary covariate set with fasting glucose or D-dimer added separately to limit model complexity.
